# Decitabine-primed tandem CD19/CD22 CAR-T therapy in relapsed/refractory diffuse large B-cell lymphoma patients

**DOI:** 10.3389/fimmu.2022.969660

**Published:** 2022-08-17

**Authors:** Changju Qu, Rui Zou, Peng Wang, Qian Zhu, Liqing Kang, Nana Ping, Fan Xia, Hailing Liu, Danqing Kong, Lei Yu, Depei Wu, Zhengming Jin

**Affiliations:** ^1^ National Clinical Research Center for Hematologic Diseases, Jiangsu Institute of Hematology, The First Affiliated Hospital of Soochow University, Suzhou, China; ^2^ Institute of Blood and Marrow Transplantation, Collaborative Innovation Center of Hematology, Soochow University, Suzhou, China; ^3^ Department of Pharmacy, The First Affiliated Hospital of Soochow University, Suzhou, China; ^4^ School of Chemistry and Molecular Engineering, East China Normal University, Shanghai, China; ^5^ Shanghai Unicar-Therapy Bio-medicine Technology Co., Ltd., Shanghai, China; ^6^ Department of Radiology, People’s Hospital of Binhai County, Binhai Cinlical College of Yangzhou University, Yancheng, China

**Keywords:** chimeric antigen receptor T cells, relapsed/refractory, diffuse large B-cell lymphoma, decitabine, complete remission

## Abstract

Chimeric antigen receptor T cell (CAR-T) therapy has emerged as highly effective in relapsed/refractory (R/R) diffuse large B-cell lymphoma (DLBCL), but only about 40% patients have achieved sustained responses. Here, we conducted a phase II clinical trial testing efficacy and toxicities of CAR-T therapy in R/R non-Hodgkin’s lymphoma patients (NCT03196830). Among enrolled patients, 33 R/R DLBCL patients pretreated with DFC (decitabine, fludarabine plus cyclophosphamide) lymphodepletion chemotherapy and infused with tandem CD19-CD22 based CAR-T cells were drawn out for efficacy and toxicities of CAR-T therapy evaluation. With a median follow-up of 10.9(0.6-29.0) months, the best overall response and complete remission (CR) rates were 90.9% and 63.6%, respectively. The median progression-free survival (PFS) was 10.2 months and overall survival (OS) was undefined. The 2-year OS and PFS rates were 54.3% and 47.2%, respectively. No severe grade 4 cytokine release syndrome (CRS) was observed and grade 3 CRS was observed in only 7 patients; 3 patients developed mild immune effect or cell-associated neurotoxic syndrome. All toxicities were transient and reversible and no CAR-T-related mortality. Further subgroup analysis showed that achieving CR was an independent prognostic factor associated with favorable PFS and OS. The 2-year OS and PFS for patients who achieved CR within 3 months (undefined versus undefined *P*=0.021 and undefined versus undefined *P*=0.036) or during the follow-up period were significantly longer than those who did not (undefined versus 4.6 months *P* < 0.0001 and undefined versus 2.0months *P*<0.001). While severe CRS was also an independent prognostic factor but associated with inferior PFS and OS. The 2-year OS and PFS for patients with grade 3 CRS were significantly shorter than those with grade 0-2 CRS (4.1 months versus undefined *P*<0.0001 and 1.7 months versus undefined *P*=0.0002). This study indicated that CD19/CD22 dual-targeted CAR-T therapy under a decitabine-containing lymphodepletion regimen may be a safe, potent effective approach to R/R DLBCL patients.

## Introduction

Chimeric antigen receptor T cell (CAR-T) therapy representes a potentially curative approach to diffuse large B-cell lymphoma (DLBCL) patients; however, only about 40% of patients achieve sustained responses (1, 2). High relapse rate, due to antigen escape, uncertainties surrounding the cell-intrinsic property and exhaustion of CAR-T cells, has restricted CAR-T efficacy and its widespread clinical application.

To overcome antigen escape, bispecific CAR-T therapy simultaneously targeting both CD19 and CD22 is an option. Wei G et al. found that CD19/CD22 dual-targeted CAR-T therapy was effective in R/R aggressive B-cell lymphoma with 14(87.5%) achieving objective response and 10 (62.5%) achieving complete response (CR) among 16 eligible patients. Further, bispecific CAR-T therapy was also safe with only 1 patient presenting with severe cytokine release syndrome (CRS) and no patients developing immune effect or cell-associated neurotoxic syndrome (ICANS). The 2-year overall survival (OS) and progression-free survival (PFS) rates were 77.3% and 40.2%, respectively (3).

Abnormal hypermethylations lead to tumor suppressor genes silencing which are often observed in lymphoma patients. Decitabine(DAC), a DNA demethylating agent, has been reported to confer to demethylation of tumor suppressor genes and result in tumor cell growth inhibition (4–6). Moreover, Li et al. (7) demonstrated that DAC, in addition to its demethylation effect directly on lymphoma cells, can also upregulate CD19 expression on lymphoma cells and enhance the CAR-T cell-specific killing function *in vitro*. Two relapsed and refractory (R/R) lymphoma patients who underwent a combination of DAC and CAR-T therapy achieved complete remission (CR) and remained disease-free for 4 months and 2 months respectively. Further, DAC has a very limited impact on CAR-T cell viability, proliferation, and cytolytic functions. These studies provide a strong rationale for the combination of DAC and CAR-T therapy in R/R DLBCL. However, the optimal time of DAC application during CAR-T therapy including before, concomitantly with, or after CAR-T infusion is still needed to further exploit.

In this study, we reported the institutional experience at the First Affiliated Hospital of Soochow University from 33 decitabine-containing lymphodepletion regimens pretreated and tandem CD19/CD22 CAR-T infused R/R DLBCL patients drawn out from one ongoing phase II trial testing efficacy and toxicities of CAR-T therapy in R/R Non-Hodgkin lymphoma patients (http://ClinicalTrials.gov NCT03196830). Our data demonstrated that DAC-primed CD19/CD22 dual-targeted CAR-T therapy may be a safe, potent effective approach to R/R DLBCL patients.

## Materials and methods

### Patients and study design

The phase II clinical trial ran from June 23, 2017 through now at the First Affiliated Hospital of Soochow University. 33 R/R DLBCL patients received lymphodepletion chemotherapy with DFC regimen (DAC a total dose of 100mg/m^2^ was equally intravenously administrated for 3 consecutive days, cyclophosphamide 300mg/m^2^×3d and fludarabine 30mg/m^2^×3d). Two days after chemotherapy, tandem CD19/CD22 based CAR-T cells at a total dose of 1×10^7^ cells per kilogram were infused within 3 days by escalation (10%, 30%, and 60% of total dose). CAR-T efficacy and safety were evaluated. A follow-up to evaluate the duration of response, survival, and late adverse events was ongoing. The final follow-up visit for endpoint analysis was conducted on April 1, 2022. Patients’ characteristics, toxicities and responses to CAR-T were shown in **Table 1**.

**Table 1:** Baseline characteristics of patients.

Characteristics		n (%)
Age (year)		
	Median (range), years	55(31-72)
Gender		
	Male	17(51.5)
	Female	16(48.5)
Primary Diagnose		
	GCB	13(39.4)
	Non-GCB	20(60.6)
Stage		
	I/II	6(18.2)
	III/IV	27(81.8)
Status before leukapheresis		
	PR	11(33.3)
	SD	1(3)
	PD	21(63.7)
Prior lines of therapy before leukapheresis		
	1	2(6.1)
	2	11(33.3)
	3	13(39.4)
	4	4(12.1)
	>4	3(9.1)
Refractory/relapse		
	Refractory	26(78.8)
	Relapsed	7(21.2)

GCB for germinal center B cell; PR or partial response; SD for steady disease; PD for progression disease.

### CAR-T cells preparation

The peripheral blood lymphocytes were acquired and collected through density gradient separation from patients’ peripheral blood. T lymphocytes were further isolated by degradable anti-CD3 magnetic microbeads (Miltenyi Biotec, Bergisch Gladbach, Germany) and activated with 5ug/mL monoclonal anti-CD3/CD28 antibodies(Miltenyi Biotec, Bergisch Gladbach, Germany) for 48 hours. Then T cells were transduced with lentivirus encoding the CD19/22-4-1BB-CD3z transgene as previously reported (8) and cultured in AIM-V media (Gibco, NY, USA) supplemented with 10% autologous serum, 100 IU/ml IL-2, 5 ng/ml IL-7, and 5 ng/ml IL-15 for 12~20 days until their numbers met the pre-set value. All these CAR-T products were provided by the unicar-therapy bio-medicine technology co.(Shanghai, China). Quality tests were performed before infusion to patients as previously described (9–11).

### End points and assessments

The primary endpoint was overall response rate [ORR, calculated as the combined rates of CR and partial response (PR)], as assessed by the investigators according to the International Working Group Response Criteria for Malignant Lymphoma (12). Secondary endpoints included the duration of response, the incidence of adverse events (AEs), and the survival of CAR-T cells detected in patients’ peripheral blood. AEs were evaluated according to the Common Terminology Criteria for Adverse Events, version 4.03, set by the U.S. Department of Health and Human Services. CRS was graded according to the criteria of reported (13). Secondary endpoints included OS calculated from the day of CAR-T infusion to death or the end of follow-up and PFS calculated from the day of CR after CAR-T therapy to relapse or death, or the end of follow-up. CAR-T cell expansions were analyzed as described previously (9–11).

### Statistics

Analyses were performed using Graphpad Prism 5. The Kaplan-Meier approach was performed to estimate time-to-event analyses. Characteristics, efficacy and safety analyses in two cohorts were assessed with two-sided Student’s t-tests or Fisher’s exact test. Changes in cytokines and CAR-T copies in two cohorts were analyzed by two-way ANOVA. *P* values of less than 0.05 were considered statistically significant.

### Study approval

This study was approved by the Ethics Committee of the First Affiliated Hospital of Soochow University and was conducted in accordance with the Declaration of Helsinki principles. All patients provided written informed consent.

## Results

### Demographics and baseline characteristics

**Figure 1:**
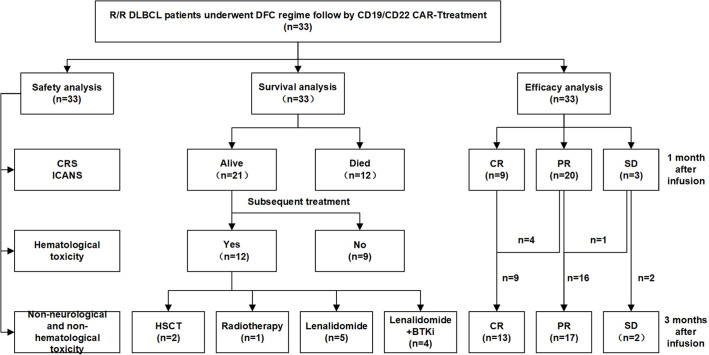
Flow chart of treatments of the enrolled 33 R/R DLBCL patients. .

**Figure 2:**
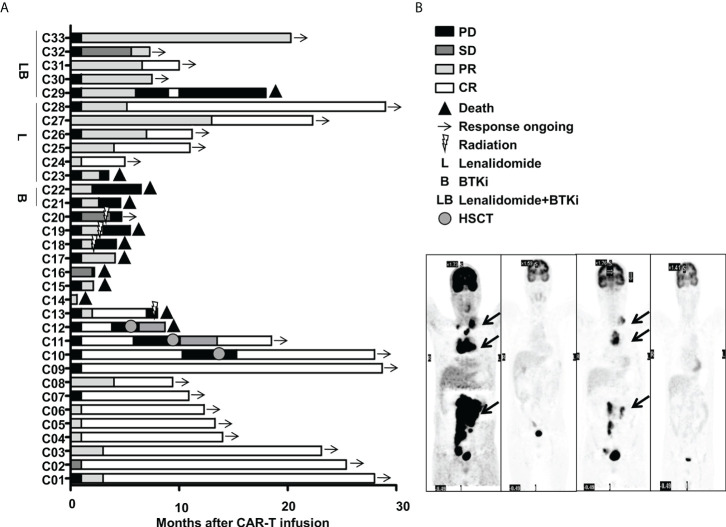
Clinical efficacy after CAR-T therapy in 33 R/R DLBCL patients. **(A)** Duration of response and survival post-infusion with CAR-T cells in all patients. **(B)** Representative PET-CT before CAR-T infusion, 1 month after CAR-T infusion, before HSCT and 3 months after HSCT in patient C10 was shown. The black arrow points to the location of the tumor. .

This cohort included 16 females and 17 males. The median age was 55(31~72) years old. 33 R/R DLBCL patients enrolled in this study were evaluated. **Table 1** described the subject-, disease- and CAR-T-related variables of this cohort. The majority of patients were non-germinal center B cell (GCB) subtype per the Hans algorithm (14) and had Stage III or IV DLBCL before treatments. Most patients received two to four lines of pre-trial chemotherapy. Further, most patients were refractory and at progression disease (PD) status before CAR-T infusion. Within three months,13 cases (Patient C1-C13) achieved CR with 9 cases maintaining CR and 4 relapsed. Among the 4 relapsed cases, 1 was exposed and irresponsive to radiotherapy and died of PD shortly; 3 cases were exposed and responsive to allogeneic hematopoietic stem cell transplantation(allo-HSCT) with 2 achieving CR and alive and 1 achieving PR and died of severe graft versus host disease (GVHD). Furthermore, 16(Patient C18-C33) out of 20 cases without achieving CR within three months were bridged to maintenance therapies including 6 cases with lenalidomide, 2 cases with Bruton tyrosine kinase inhibitor (BTKi), 5 cases with lenalidomide and BTKi, and 3 cases with radiotherapy (**Figures 1**, **2A**).

### Safety

**Figure 3:**
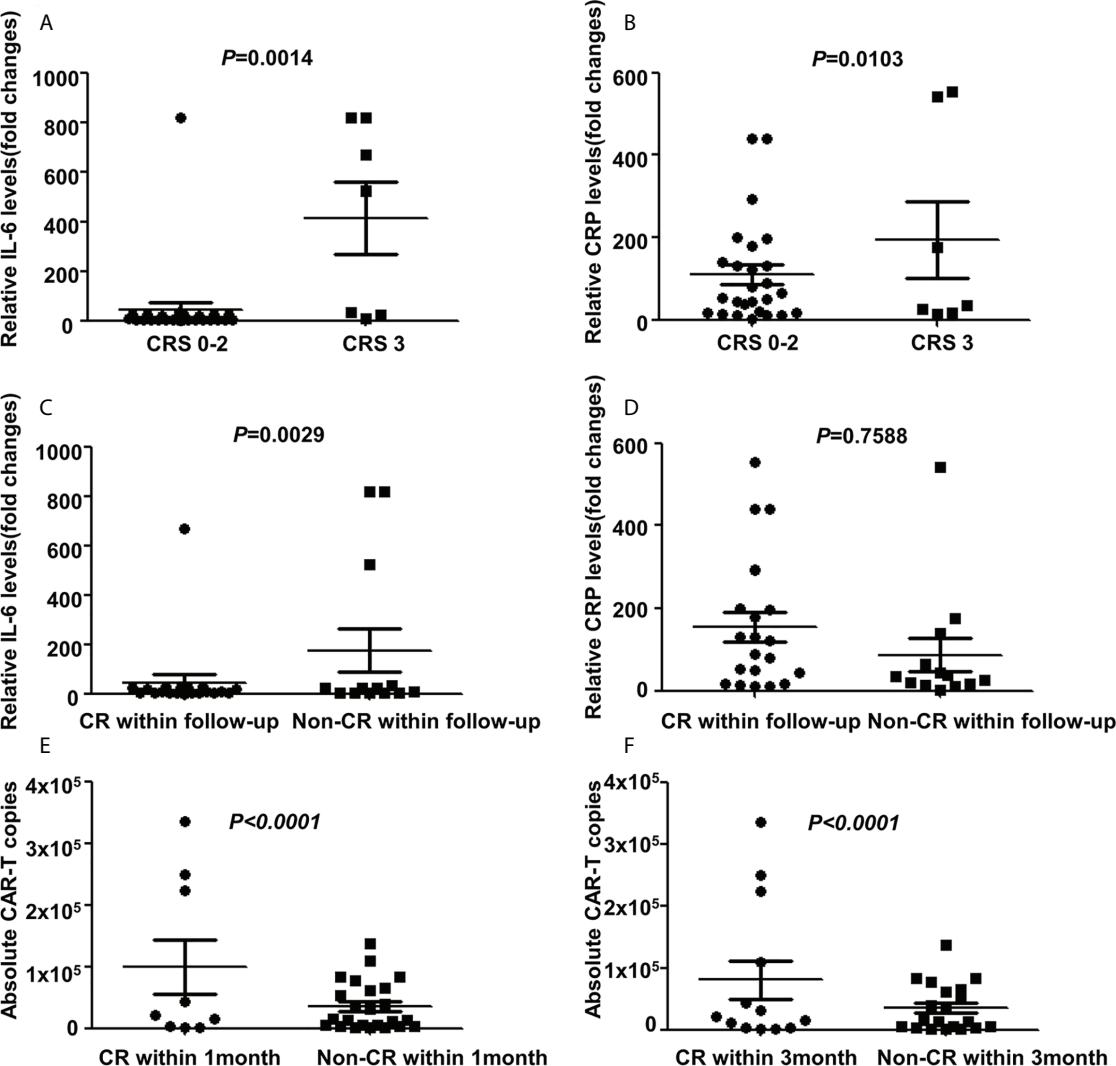
Cytokines and CAR-T copies in 33 R/R DLBCL patients. **(A, B)** Changes of IL-6 and CRP levels in patients with different CRS grades after CAR-T therapy. Significant higher IL-6 and CRP levels were detected in cases with grade CRS 3 than in that cases with grade CRS 0-2 (*P*=0.0014 and *P*=0.0103 respectively two-way ANOVA, shown are means±SD; n1=7, n2=26). **(C, D)** Changes of IL-6 and CRP levels in patients with different responses after CAR-T therapy. Patients achieving CR during follow-up period has a lower IL-6 increase than patients without achieving CR (*P*=0.0029, two-way ANOVA, shown are means±SD; n1= 20, n2=13), while CRP had no difference in both groups(*P*=0.7588, two-way ANOVA, shown are means±SD; n1=20, n2=13). **(E, F)** Changes of CAR-T copies in patients with different response after CAR-T therapy. Relatively higher CAR-T copies were observed in cases that achieved CR than cases without CR within 1 month(*P<0.0001*, two-way ANOVA, shown are means±SD; n1=9, n2=24) or 3 months(*P<0.0001*, two-way ANOVA, shown are means±SD; n1=13, n2=20). Note: CR for complete remission; CRS for cytokine release syndrome; IL-6 for Interferon-6; CRP for c-reactive protein.

All AEs occurring within 14 days of CAR-T cells infusion were graded and reported for the 33(100%) treated patients. The most frequent AEs were severe hematological toxicities. Grade III/IV neutropenia was observed in all 33 cases (100%), severe anemia, thrombocytopenia and hemagglutination abnormalities were observed in 23(69.7%), 30(90.9%) and 12(36.4%) cases respectively. CRS was observed in 25(75.8%) patients included grade 3 CRS (21.2%), grade 2 CRS (12.1%) and grade 1 CRS (42.4%) respectively. Patients with mild CRS (grade 0-2) responded to supportive care, while patients with severe CRS (grade 3), 3 cases were resolved with tocilizumab and 4 cases were controlled by rapid steroid taper besides supportive care. Further, 3 patients (9.1%) developed ICANS who also manifested with severe CRS. After administration of steroid, ICANS were reversed quickly. A panel of cytokines relevant to CAR-T therapy were detected with a dramatically higher increase of interferon (IL)-6 and c-reactive protein (CRP) levels in cases with grade 3 CRS than in other cases (*P*=0.0014 and *P*=0.0103 respectively, **Figures 3A, B**). Further analysis showed that patients achieving CR during the follow-up period has a lower IL-6 increase than patients without achieving CR (*P*=0.0029, **Figure 3C**), while the CRP had no difference in both groups (*P*=0.7588, **Figure 3D**). Non-neurological and non-hematological toxicities included pyrexia (78.8%), hypoxia (36.4%), hypotension (3.0%), cardiac failure (6.0%), acute kidney injury (15.2%), pneumonia (12.1%), increase of alanine transaminase (24.2%) and hyperbilirubinemia (3.0%). Generally, all cases were well-tolerated and all toxicities were transient and reversible and no CAR-T-related mortality (**Table 2**).

**Table 2:** Toxicities after CAR-T therapy in all patients.

Toxicities		n (%)
Cytokine-release syndrome grade		
	0	8(24.2)
	1-2	18(54.5)
	3-5	7(21.2)
Immune effect or cell associated neurotoxic syndrome		3(9.0)
Hematological toxicity		
	Neutropenia (III/IV)	33(100)
	Anemia (III/IV)	23(69.7)
	Thrombocytopenia (III/IV)	30(90.9)
	Hemagglutination abnormalities(III/IV)	12(36.4)
Non-neurological and non-hematological toxicity		
	Pyrexia	26(78.8)
	Hypoxia	12(36.4)
	Hypotension	1(3.0)
	Cardiac failure	2(6.0)
	Acute kidney injury	5(15.2)
	Ascites/Hydrothorax	0(0)
	Pneumonia	4(12.1)
	Increase of alanine-aminotransferase	8(24.2)
	Increase of Hyperbilirubinemia	1(3.0)
	Tumor lysis syndrome	0(0.0)

### Efficacy and survival

As of April 1, 2022, 33 cases were enrolled and 32 cases were eligible for further efficacy evaluation as one case was discharged due to financial problems on day 11 and died on day 18 after CAR-T infusion due to pneumonia. Within 1 month and 3 months after CAR-T infusions, 29 (90.6%) and 30(93.8%) achieved an objective response with 9 (28.1%) and 13(40.6%) achieving CR. The best ORR and CR rate (CRR) were 93.8% and 63.6% respectively. Further subgroup analysis showed that the best CRR was significantly higher in cases at PR/stable disease (SD) status than cases at PD status before CAR-T infusion (90% vs 50%, *P*=0.0303), while there was no significant difference at the best ORR as well as CRR/ORR within one or three months between two groups(*P*>0.05). Among 7 patients with severe CRS, 6 were eligible for efficacy evaluation. Within 1 month or 3 months follow-up, 1 achieved CR and 5 achieved PR. The ORR and CRR were 100% and 16.7%, respectively. Among the 26 patients with mild CRS, 8 achieved CR, 15 achieved PR and 3 maintained SD within 1 month follow-up; while 12 achieved CR, 12 achieved PR and 2 maintained SD within 3 months follow-up. The ORR and CRR were 88.5% and 30.5% within 1 month follow-up and 92.3% and 46.2% within 3 months follow-up, respectively. However, there were no significant difference on the efficacy among patients with different grade of CRS. Furthermore, after CAR-T infusion, relatively high (7.8×10^3^-3.4×10^5^/ml) CAR-T copies were observed in all patients with significantly higher copies in cases achieved CR than in cases without CR within 1 month or 3 months(*P<0.0001*) (**Figures 3E, F**).

With the follow-up to date, 21 cases were still alive and 12 cases deceased. Among the 21 alive cases, 17 cases were in ongoing CR and 3 cases were in ongoing PR and 1 case was at PD. Among the 12 deceased cases, 3 cases died of pneumonia at 18 days, 2.1 months and 4.1 months after CAR-T infusion, one case died of severe GVHD 3.3months after allo-HSCT who achieved CR but relapsed 2.8 months after CAR-T and was exposed to allo-HSCT, while the other 8 cases died of PD with 7 cases within 12 months and 1 case at 18.0 months after CAR-T therapy. Among the patients with ongoing CR, one patient (C10) who responded to CAR-T therapy initially and relapsed shortly was exposed to allo-HSCT and achieved CR (**Figure 2B**). **Figure 2B** showed representative PET- CT before CAR-T infusion, 1 month after CAR-T infusion, as well as before HSCT and 3 months after HSCT in patient C10. The patient was previously treated with 4 cycles of R-CHOP (rituximab plus cyclophosphamide, doxorubicin, vincristine, and prednisone) and 2 cycles of R-GemOx (rituximab plus gemcitabine and oxaliplatin) but got PD. Then the patient was exposed to CAR-T therapy and achieved CR 1 month after CAR-T therapy, but relapsed 9.3 months after CAR-T infusion. The patient was then exposed to radiation, immunochemotherapy BR (bendamustine plus rituximab) regimen, targeted therapies including BTKi, B-cell lymphoma-2 inhibitor (BCL-2i) and chidamide, followed by allo-HSCT and achieved CR2. Now patient C10 had been in CR for more than 1 year.

**Figure 4:**
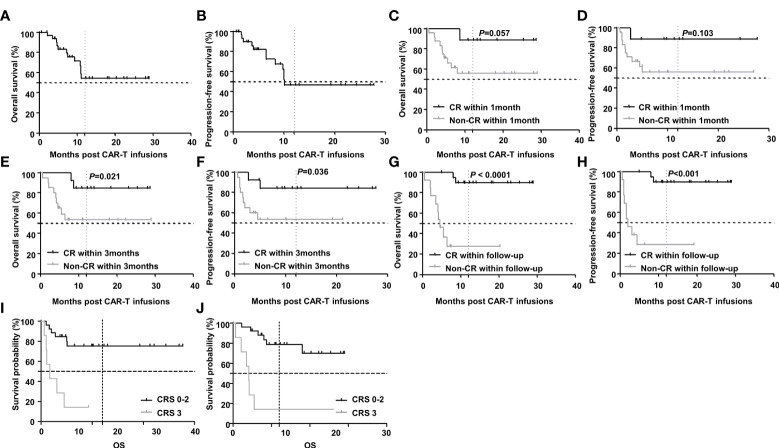
Survival after CAR-T therapy in R/R DLBCL patients. **(A, B)** OS and PFS curves of all patients after CAR-T therapy and the 2-year OS and PFS rates were 54.3% and 47.2% respectively (n=33). **(C, D)** OS and PFS curves of patients in cases who achieved CR within 1 month and those who did not. The 2-year OS and PFS in both groups were undefined and undefined (n1=9, n2=24, *P*=0.057 and *P*=0.103 respectively). **(E, F)** The 2-year OS and PFS curves of patients in cases who achieved CR within 3 months and those who did not. The 2-year OS and PFS in both groups were undefined and undefined (n1=13, n2=20, *P*=0.021 and *P*=0.036 respectively). **(G, H)** The 2-year OS and PFS curves of patients in cases who achieved CR during the follow-up period and those who did not. The 2-year OS and PFS in both groups were undefined versus 4.6 months (n1=20, n2=13, *P* < 0.0001) and undefined versus 2.0 months (n1=20, n2=13, *P*<0.001). **(I, J)** The 2-year OS and PFS curves of patients with grade 3 CRS and those with grade 0-2 CRS. The 2-year OS and PFS in both groups were 4.1 months versus undefined (n1=7, n2=26, *P*< 0.0001) and 1.7 months versus undefined (n1=7, n2=26, *P*=0.0002). Note: OS for overall survival; PFS for progression-free survival.

With a median follow-up of 10.9(0.6-29.0) months, the 2-year OS and PFS rates were 54.3% and 47.2% respectively (**Figures 4A, B**). Further subgroup analysis showed that achieving CR was an independent prognostic factor associated with favorable PFS and OS. Though there was only a trend of difference in OS and no significant difference in PFS between patients who achieved CR within 1 month and those who did not (undefined versus undefined *P*=0.057 and undefined versus undefined *P*=0.103). But the 2-year OS and PFS for patients who achieved CR within 3 months (undefined versus undefined *P*=0.021 and undefined versus undefined *P*=0.036) or during the follow-up period were significantly longer than those who did not (undefined versus 4.6 months *P* < 0.0001 and undefined versus 2.0months *P*<0.001). While severe CRS was also an independent prognostic factor but associated with inferior PFS and OS. The 2-year OS and PFS for patients with grade 3 CRS were significant shorter than those with grade 0-2 CRS(4.1 months versus undefined *P*<0.0001 and 1.7 months versus undefined *P*=0.0002). (**Figures 4C–J**).

## Discussion

This study firstly reported the safety, efficacy as well as the survival of tandem CD19/CD22 CAR-T therapy with DAC containing lymphodepletion regimen in 33 R/R DLBCL and addressed the additional benefit of DAC in CAR-T treated R/R DLBCL patients. Patients showed high efficacy, good survival and reversible toxicities highlighting that DFC may be an effective and safe lymphodepletion regimen before CAR-T therapy. However, due to the small sample size as well as the single-arm phase II trial, the results must be considered preliminary and should be verified by large-scale randomized controlled trials in the future.

It had been reported that DAC can upregulate tumor-specific antigens (7), increase the expansion of cytotoxic immune cells including CAR-T cells (7), prevent T-cell exhaustion (15), induce cell death through DNA damage-mediated G2/M or S phase arrest (16) and inhibit GVHD and enhancement of graft-versus-leukemia/lymphoma (GVL) effects after allo-HSCT (6). In our previous study, we found that additional DAC application may improve the outcome of CAR-T therapy in a high-risk population of R/R acute leukemia patients with *TP53* alterations. Moreover, it was reported that DAC enhanced CAR-T therapy in lymphoma cells *in vitro* and two R/R lymphoma patients who underwent a combination of DAC and CAR-T therapy achieved CR. However, this study only enrolled two single cases with one R/R Burkitt lymphoma and one R/R DLBCL and there was no long-term follow-up data about DAC effects on CAR-T therapy in lymphoma. The effect of DAC on DLBCL patients’ efficacy and long-term survival underwent CAR-T therapy was still not clarified and needed further investigation. Our study filled the gap in this field to some extent.

CRS and ICANS were frequent and more severe in patients with high tumor burden (6) and remained challenges to safe CAR-T therapy (17). Further, it was reported that patients with high tumor burden showed less efficacy and worse survival after CAR-T therapy. In our study, 6 cases showed grade 3 CRS and 3 cases presented ICANS and all these cases showed high tumor burden and were at PD status before CAR-T infusions. Moreover, patients at PD status before CAR-T infusion presented with less CRR and more severe CRS compared to patients at PR/PD status. These were consistent with the previous report. However, there was no significant difference in PFS or OS between the two subgroups of patients. The relatively good survival of patients at PD status may be due to the application of DAC in the lymphodepletion regimen as well as maintenance therapy after CAR-T therapy.

The main barrier to achieving a durable clinical response for CAR-T therapy was the high relapse rate due to antigen loss. To conquer relapse after CAR-T therapy, one strategy was to engineer multi-specific CAR-T cells against different potency targets (18). CD22-targeted CAR-T cells had shown an impressive response rate in DLBCL patients resistant to CD19-targeted CAR-T therapy (19, 20). Moreover, CD19/CD22 dual-target CAR-T cells had been reported to be safe and effective to reduce the rates in different clinical trials (18, 21). In this study, we performed CAR-T therapy using tandem CD19 and CD22 antigen targets and got a good response. One strategy was to bridge new therapy. Allo-HSCT had been considered as a potentially curative therapy for treating R/R DLBCL. Considering the high risk of transplant-related mortality, allo-HSCT was not preemptive until patients relapsed after CAR-T therapy. In our study, 3 cases who relapsed after CAR-T therapy were exposed to HSCT and with 2 achieving CR and alive and 1 achieving PR but died of severe GVHD. Our data supported that allo-HSCT may be a feasible salvage approach for high-risk DLBCL patients who relapsed after CAR-T therapy. One strategy was the application of external pharmacological interventions. In the literature, BTKi (22, 23), lenalidomide (24, 25), PD-1 inhibitor (26), BCL-2i (27), idelalisib (28) and interferon α (29) had been reported to synergize with CAR-T cells in the treatment of various hematologic malignancies, and these compounds may be potential therapy choices for combination therapies with CAR-T therapy. However, most studies were limited to preclinical settings and more clinical evidence was needed to confirm these approaches. In our study, 16 cases without achieving CR within three months received maintenance therapies including 6 cases with lenalidomide, 2 cases with BTKi, 5 cases with lenalidomide and BTKi, and 3 cases with radiotherapy. Finally, 5 out of 6 patients administrated lenalidomide achieved CR and alive, 5 out of 5 patients administrated lenalidomide and BTKi achieved CR with 4 cases alive except one case withdrawal of drugs unauthorized and died of PD. However, 2 cases administrated with BTKi and 3 cases exposed to radiotherapy were irresponsive and died of PD. Our study supported that lenalidomide-containing regimen may be a good maintenance approach to enhancing CAR-T efficacy for patients without achieving CR after CAR-T in DLBCL. Another strategy was to optimize the lymphodepletion regimen. Our group previously reported the data of 32 patients underwent FC lymphodepletion regimen and infused with tandem CD19/CD22 CAR-T cells owing the same design and produced by the same company (8). The ORR and CRR were 79.3% and 34.5% respectively which were lower than that reported in this present study (ORR and CRR were 90.9% and 63.6%, respectively). The 1-year OS and PFS rates were 63.3% and 40.0%, respectively in the previous study, while 2-year OS and PFS rates were 54.3% and 47.2%, respectively in this present study. Compared to patients without addition of DAC, patients with DAC-containing regimen present more superior efficacy and PFS. To access whether DAC addition brought additional hematological toxicities, we analyzed and compared the hematological toxicities of patients underwent lymphodepletion regimens with or without DAC. Among patients underwent DAC-containing lymphodepletion regimen, grade III/IV neutropenia was observed in all 33 cases(100%), severe anemia and thrombocytopenia were observed in 23(69.7%) and 30(90.9%), respectively. While among patients underwent no DAC-containing lymphodepletion regimen, grade III/IV neutropenia was observed in 26(81.3%) cases, severe anemia and thrombocytopenia were observed in 18(56.3%) and 17(53.1%) cases, respectively. It seemed that the hematological toxicities were more frequent after addition of decitabine. However, all hematological toxicities were transient and reversible. Our data suggested that the addition of DAC in a regular FC lymphodepletion regimen before CAR-T therapy may be a feasible and safe approach to overcome relapse after CAR-T therapy in R/R DLBCL. However, due to the small sample size as well as unbalanced patients’ characteristics between two studies, large-scale randomized controlled phase III trial should be guaranteed in the future to figure out benefit of DAC as a part of lymphodepletion regimen before CAR-T infusion.

In summary, this study represented the first successful phase II trial of using DAC containing lymphodepletion regimen DFC in R/R DLBCL. Moreover, this study demonstrated that the addition of DAC in the traditional lymphodepletion FC regimen may be a safe and promising approach to managing R/R DLBCL patients. This study provided a rationale for combining DAC and CAR-T therapy in R/R DLBCL.

## Data availability statement

The original contributions presented in the study are included in the article/supplementary material. Further inquiries can be directed to the corresponding authors.

## Ethics statement

The studies involving human participants were reviewed and approved by the Ethics Committee of the First Affiliated Hospital of Soochow University. The patients/participants provided their written informed consent to participate in this study. Written informed consent was obtained from the individual(s) for the publication of any potentially identifiable images or data included in this article.

## Author contributions

CQ, DW, and ZJ contributed to conception and design of the study. CQ, PW and QZ treated the patients and collected the clinical data. RZ and CQ organized all the data and performed the statistical analysis. LK and LY engineered and performed the quality tests of the CAR-T cells. HL performed all imaging interpretation. CQ wrote the first draft of the manuscript. NP, FX and DK wrote sections of the manuscript. All authors contributed to manuscript revision, read, and approved the submitted version.

## Funding

This work was supported by research grants from the National Key R&D Program of China (2016YFC0902800) (to DW), Priority Academic Program Development of Jiangsu Higher Education Institutions (PAPD) (to DW), National Natural Science Foundation of China [(81400155) (to CQ and ZJ), (81600114)(to NP)], Jiangsu Natural Science Foundation of China (BK20140374)(to CQ and ZJ), Top-notch young health talents, 5th Suzhou health professionals program (GSWS2019035)(to CQ), and National Clinical Research Center for hematologic disease[(2021ZKMC01)(to CQ), [(2021ZKMB03)(to NP)].

## Acknowledgments

We would like to thank Dr. Robert Gale for reviewing our manuscript. We also thank all members of the study team, the patient and their family.

## Conflict of interest

Authors LY and LK are employed by Shanghai Unicar-Therapy Bio-medicine Technology Co., Ltd.

The remaining authors declare that the research was conducted in the absence of any commercial or financial relationships that could be construed as a potential conflict of interest.

## Publisher’s note

All claims expressed in this article are solely those of the authors and do not necessarily represent those of their affiliated organizations, or those of the publisher, the editors and the reviewers. Any product that may be evaluated in this article, or claim that may be made by its manufacturer, is not guaranteed or endorsed by the publisher.
